# Variable chlorhexidine MICs across *Klebsiella* species from a single facility

**DOI:** 10.1128/aac.01436-25

**Published:** 2026-02-09

**Authors:** David Lehman, Aubrey E. Hetzler, Tayloe Friedrich, Madeline M. Strouse, Katie E. Barry, Shireen M. Kotay, Amy J. Mathers

**Affiliations:** 1 Division of Infectious Diseases and International Health, Department of Medicine, University of Virginia School of Medicine 12349, Charlottesville, Virginia, USA; 2 Clinical Microbiology, Department of Pathology, University of Virginia School of Medicine 12349, Charlottesville, Virginia, USA; Shionogi Inc., Florham Park, New Jersey, USA

**Keywords:** Chlorhexidine tolerance, Hospital environment, Carbapenemase producing Enterobacterales, *Klebsiella quasipneumoniae*

## Abstract

We assessed *in vitro* chlorhexidine minimal inhibitory concentrations (MICs) across *Klebsiella* species from a hospital with widespread chlorhexidine use. Isolates underwent MIC testing and whole genome sequencing. Species showed varying resistance, with *Klebsiella quasipneumoniae* having the highest MICs. There was no clear link to acquired resistance, but some species had more chromosomal efflux pumps. Differences in chlorhexidine MICs between species highlight the role that biocides could have in shaping microbial populations in the hospital environment.

## INTRODUCTION

The genus *Klebsiella* encompasses diverse species occupying both clinical and environmental niches. *Klebsiella pneumoniae sensu stricto* has emerged as one of the most consequential multidrug-resistant pathogens worldwide, with carbapenemase-producing *K. pneumoniae* representing an urgent public health threat and a major cause of hospital-associated infections in vulnerable patients (1). Hospital wastewater systems, including sink drains, P-traps, and toilets, are increasingly recognized as persistent reservoirs for carbapenemase-producing Enterobacterales and provide a niche for carbapenemase gene exchange, including among *Klebsiella* sp. (2, 3).

Species-level identification within the *K. pneumoniae* complex, including *K. pneumoniae sensu stricto*, *Klebsiella variicola*, and *Klebsiella quasipneumoniae*, is problematic using standard clinical microbiology methods and often requires whole-genome sequencing for accurate classification (4, 5). Although species across the *K. pneumoniae* complex have been documented to cause infections, their natural niches and virulence vary (6). Despite their prevalence in both patient and environmental reservoirs, the ecological and selective forces shaping species distribution within the hospital setting remain poorly defined, but carbapenemase gene exchange can occur between species (7, 8).

Chlorhexidine is widely used in healthcare as a patient skin antiseptic, hand hygiene agent, and equipment disinfectant (9, 10). While full clinical resistance (i.e., no longer able to achieve killing at topical concentrations) at clinically applied concentrations is unlikely for any Enterobacterales (11), interspecies variation in minimal inhibitory concentrations (MICs) has been described, and selective pressure from antiseptic use is a longstanding concern in infection control (12, 13). Outbreaks associated with *Serratia marcescens*, a species with intrinsic chlorhexidine elevated MICs, underscore the potential for disinfectants to influence species persistence and selection within hospital environments (11, 12). Whether such selective effects extend across the *Klebsiella* genus remains unclear, despite their importance as environmental and clinical reservoirs of antimicrobial resistance. This study leveraged whole-genome sequencing to accurately speciate *Klebsiella* isolates collected from environmental reservoirs and patients. Our goal was to evaluate whether chlorhexidine MICs vary across the *Klebsiella* genus within a single hospital.

Isolates were previously collected from the University of Virginia, where all intensive care unit hand hygiene soap is SCRUB-STAT (Ecolabs, St. Paul, MN) with 2% chlorhexidine gluconate. We used previously sequenced isolates across species enriched for *bla*_KPC_-positive *Klebsiella* sp. isolates from patients and the environment between 2007 and 2024. We also used several known species from curated strains from the American Type Culture Collection (ATCC Manassas, VA) as species controls where available. Chlorhexidine was diluted in sterile water and then applied to Cation-Adjusted Müeller Hinton Broth (CAMHB), with final concentrations ranging from 0.125 μg/mL to 128 μg/mL in doubling dilutions using the methods described in Lutgring et al. (14). To achieve the highest test concentration of 128 μg/mL without precipitation, an increase from 64 μg/mL previously described, both CAMHB and chlorhexidine solutions were pre-warmed to 37°C prior to combining. All isolates were tested in triplicate. All isolates underwent whole-genome sequencing using a MiSeq v2 300 cycle Reagent Kit on the MiSeq platform (Illumina, San Diego, CA), and some additional isolates had also previously undergone long read sequencing using PacBio or Oxford Nanopore as previously described (15, 16). Speciation and strain typing were performed by calculating the genomic distances of the read sets against a curated collection of complete bacterial chromosomal assemblies in National Center for Biotechnology Information (NCBI) RefSeq database using MASH (17) (v2.1). *De novo* assembly was checked against the AMRFinder database in NCBI with >99% identity and coverage. Isolates were selected to capture a variety of species within the genus *Klebsiella* and to compare them with *Escherichia coli*, which serves as a standard, and *S. marcescens*, known to exhibit higher MICs (18). To evaluate differences in log2-transformed MIC values among bacterial species, we first conducted a Kruskal-Wallis test to assess overall group differences (α = 0.05). Post-hoc pairwise MICs between species with comparisons using Dunn’s test, implemented from the rstatix R package (v. 0.7.2), with a Bonferroni correction.

We tested 78 unique isolates; 57 clinical, 15 environmental, and 6 ATCC against chlorhexidine (Table 1). The technique had good reproducibility with all replicates being within one dilution, thus 100% essential agreement across the triplicate testing with the median used as the MIC.

**TABLE 1:** Isolate collection and chlorhexidine MIC testing results

	MIC50 µg/mL	MIC90 µg/mL	Range µg/mL	Clinical	Standard^*b*^	Environmental
*K. pneumoniae sensu stricto* *n* = 13	16	32	8–32	10	3	0
*K. quasipneumoniae* *n* = 22	32	64	16–64	10	1	11
*Klebsiella oxytoca* *n* = 3	8	16	4–16	2	0	1
*Klebsiella aerogenes* *n* = 6	32	32	16–32	6	0	0
*Klebsiella grimondii^a^* *n* = 1	N/A	N/A	32–32	0	0	1
*K. variicola* *n* = 5	32	32	16–32	5	0	0
*Klebsiella michiganensis* *n* = 4	16	32	4–32	3	0	1
*E. coli* *n* = 9	2	8	1–32	7	1	1
*S. marcescens* *n* = 15	128	128	32–128	14	1	0

^*a*^ Not applicable (N/A) as no calculation of MIC50/90 with a single isolate.

^*b*^ American Type Culture Collection (ATCC).

**Fig 1:**
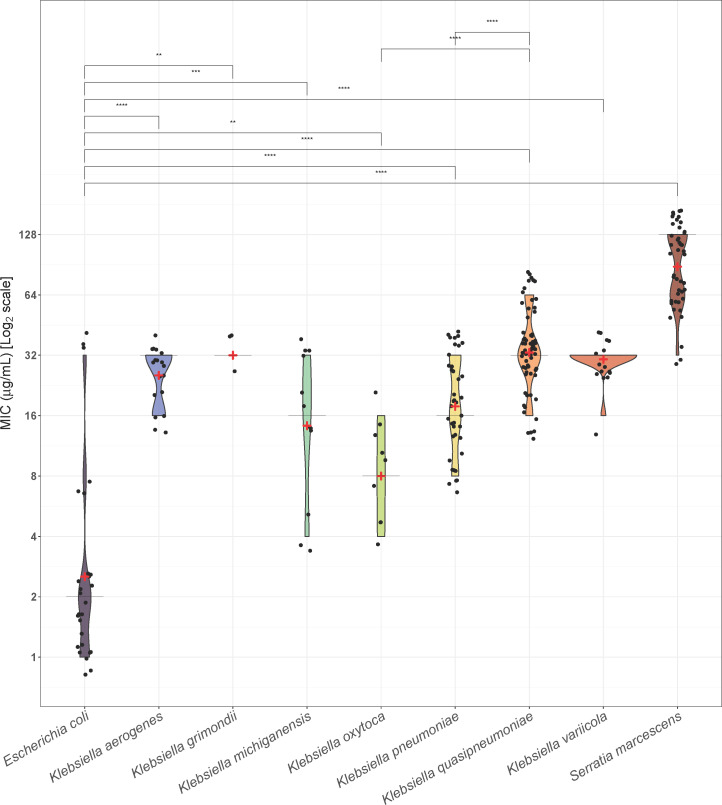
Violin plots depicting MIC distribution for chlorhexidine by species. Red plus signs indicate the mean log_2_(MIC) concentration for each species, while black horizontal bars represent the median values. A Kruskal-Wallis test indicates an overall statistically significant difference in MIC distributions across species.The bars represent pairwise comparisons using a Wilcox Test. **: *P* ≤ 0.01, ***: *P* ≤ 0.001, ****: *P* ≤ 0.0001.

In our study, like others, *E. coli* generally exhibited lower MICs compared to all *Klebsiella* species, including *K. pneumoniae* (*P* = 0.02), *Klebsiella aerogenes* (*P* = 0.001), *K. quasipneumoniae*, *K. variicola*, and *S. marcescens* (all *P*<0.0001) but not *Klebsiella grimondii, Klebsiella oxytoca,* and *Klebsiella michiganensis*, although numbers are small (Fig. 1, Table S2). A larger prior evaluation showed *E. coli* had an MIC₉₀ of 16 µg/mL, whereas *K. pneumoniae* demonstrated an MIC₉₀ of 32 µg/mL (19). Interestingly, in this collection, several *K. quasipneumoniae* isolates displayed higher MICs, with an MIC_50_ of 32 and MIC₉₀ of 64 µg/mL. Using the Dunn statistical test, MICs of *K. quasipneumoniae* were significantly higher than *K. oxytoca* (*P* = 0.002) and *K. pneumoniae sensu stricto* (*P* = 0.004).

We further evaluated resistance gene content across this multidrug-resistant collection using AMRFinder (NCBI). Predicted genes included quaternary ammonium compounds (qac) determinants that may be plasmid borne and drive transferable efflux in gram positives, but these were not consistently found, nor was there a clear correlation with chlorhexidine MICs (Fig. S1 and S2). Previous studies in gram-negative bacilli have failed to demonstrate that mobile AMR genes promote chlorhexidine resistance conclusively but rather the elevated MICs are more likely selected by chromosomal genes and mechanisms allowing resistance to killing, such as outer membrane differences and effective efflux pumps (20, 21). Interestingly, both *the K. variicola* and *K. quasipneumoniae* consistently had resistance nodulation cell division family oqxB efflux pumps, as did several of the *K. pneumoniae* (22). In terms of gene loci, across the 32 isolates with long-read assemblies, all *oqx* genes were chromosomally encoded in *Klebsiella* species, *tet(41*) was consistently chromosomal in *Serratia,* and in contrast, all qac genes, *tet(A*) and *tet(B),* when present were located on plasmids across several species and genera (Table S3).

At our institution, where chlorhexidine-impregnated soap is routinely used, we have found ongoing recovery of *bla*_KPC_-positive isolates from hospital wastewater plumbing, particularly sink drains (23). Sequencing has revealed that several isolates initially identified as *K. pneumoniae* were further speciated via sequencing as *K. quasipneumoniae* (8). We have also frequently detected *bla*_KPC_-positive *S. marcescens* in this plumbing reservoir, and a strain that has been seen in patients and the environment within our hospital was included (CAV1492) (24).

These preliminary findings suggest a potential link between environmental persistence, biocide resistance to killing, and antimicrobial resistance. However, the small sample size and the selection from a single institution mean the data should be viewed as very preliminary. Variation within the genus *Klebsiella* may reflect adaptation to distinct ecological niches and contribute to environmental persistence (25). Consistent with this, species more commonly linked to environmental reservoirs, including *K. variicola* and *K. quasipneumoniae*, exhibited higher chlorhexidine MICs. This raises the possibility that biocide exposure in healthcare or environmental settings is selecting for strains with both increased MICs to disinfectants and the ability to carry clinically relevant resistance determinants such as *bla*_KPC_ driving outbreaks related to premises plumbing (26, 27).

Although the data set is limited in size, it highlights the importance of species-level identification and emphasizes the need to consider both clinical and environmental isolates in infection control strategies. The observed differences in chlorhexidine MICs between species underscore the role of ecological pressures in shaping microbial populations. Co-selection of antimicrobial resistance through biocide exposure may be an underappreciated factor in the emergence and spread of multidrug-resistant organisms in healthcare settings, warranting further investigation into the mechanisms of resistance to biocide killing and their epidemiological consequences.

## Data Availability

All Next Generation Sequencing data included in this study have been deposited in the NCBI Sequence Read Archive under BioProject ID- PRJNA246471, PRJNA353060, PRJNA411762, PRJNA1170947, PRJNA1418706.

## SUPPLEMENTAL MATERIAL

Supplemental material aac.01436-25-s0001.docx Fig. S1 and S2; Tables S1 to S3.

ASM does not own the copyrights to Supplemental Material that may be linked to, or accessed through, an article. The authors have granted ASM a non-exclusive, world-wide license to publish the Supplemental Material files. Please contact the corresponding author directly for reuse.
