# Supplementary material for: Variable chlorhexidine MICs across Klebsiella species from a single facility
Source: Antimicrob Agents Chemother. 2026 Feb 9;70(3):e01436-25. doi: 10.1128/aac.01436-25 (PMC12959085; doi:10.1128/aac.01436-25)
Supplement: Supplemental material — Fig. S1 and S2; Tables S1 to S3. [file aac.01436-25-s0001.docx]

Supplement

Figure S1. AMR Gene profiles for each isolate of *Klebsiella* sp. grouped by species as identified by AMRFinder with >99% identify and coverage. AMR gene color coded by drug class. Log transformed MIC mode value for each corresponding isolate represented as bar plots. Sorted in ascending order of MIC mode. Mean MIC value for each species represented as horizontal line


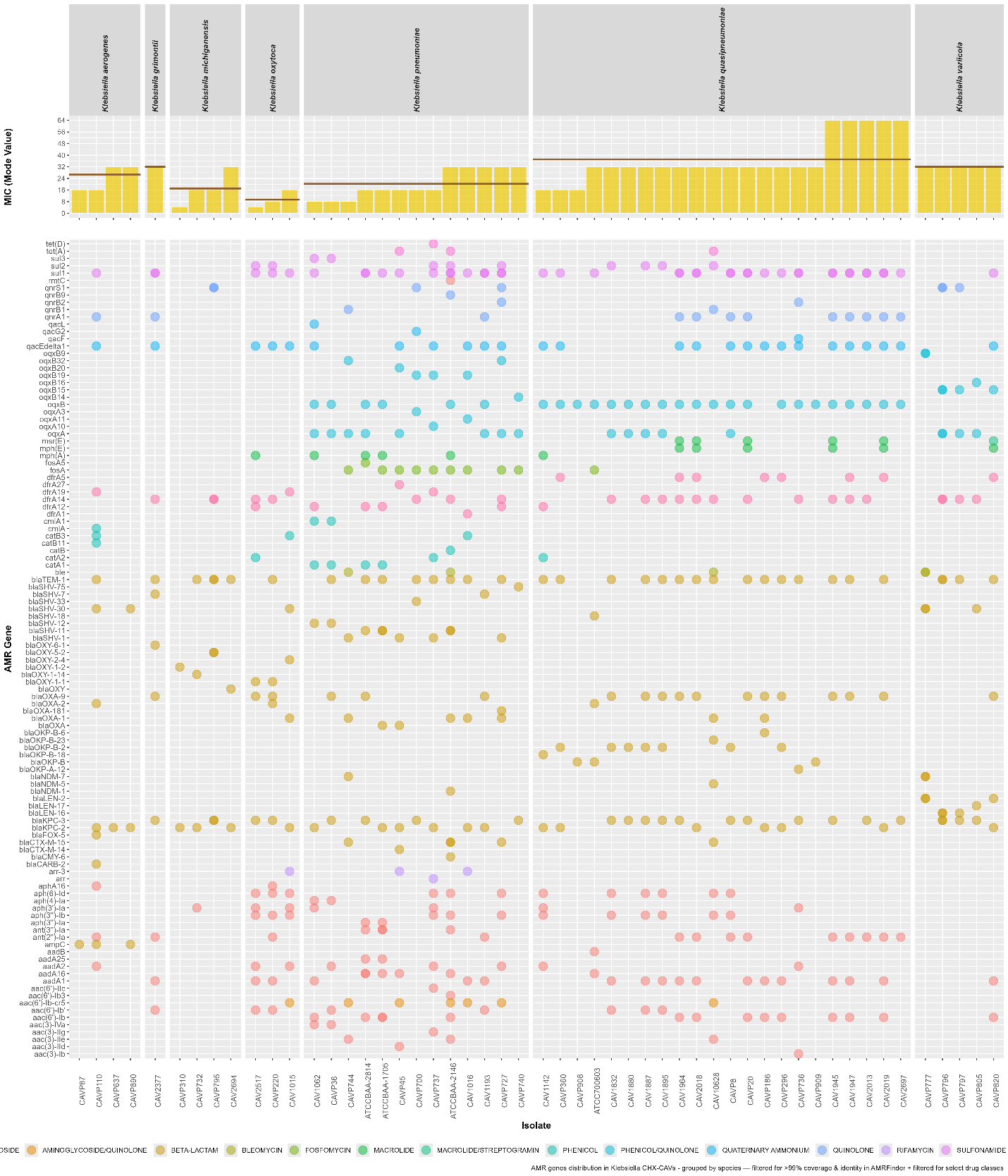


Figure S2. AMR Gene profiles for each isolate of *E. coli and S. marcescens* as identified by AMRFinder with >99% identify and coverage. AMR gene color coded by drug class. Log transformed MIC mode value for each corresponding isolate represented as bar plots. Sorted in ascending order of MIC mode. Mean MIC value for each species represented as horizontal line


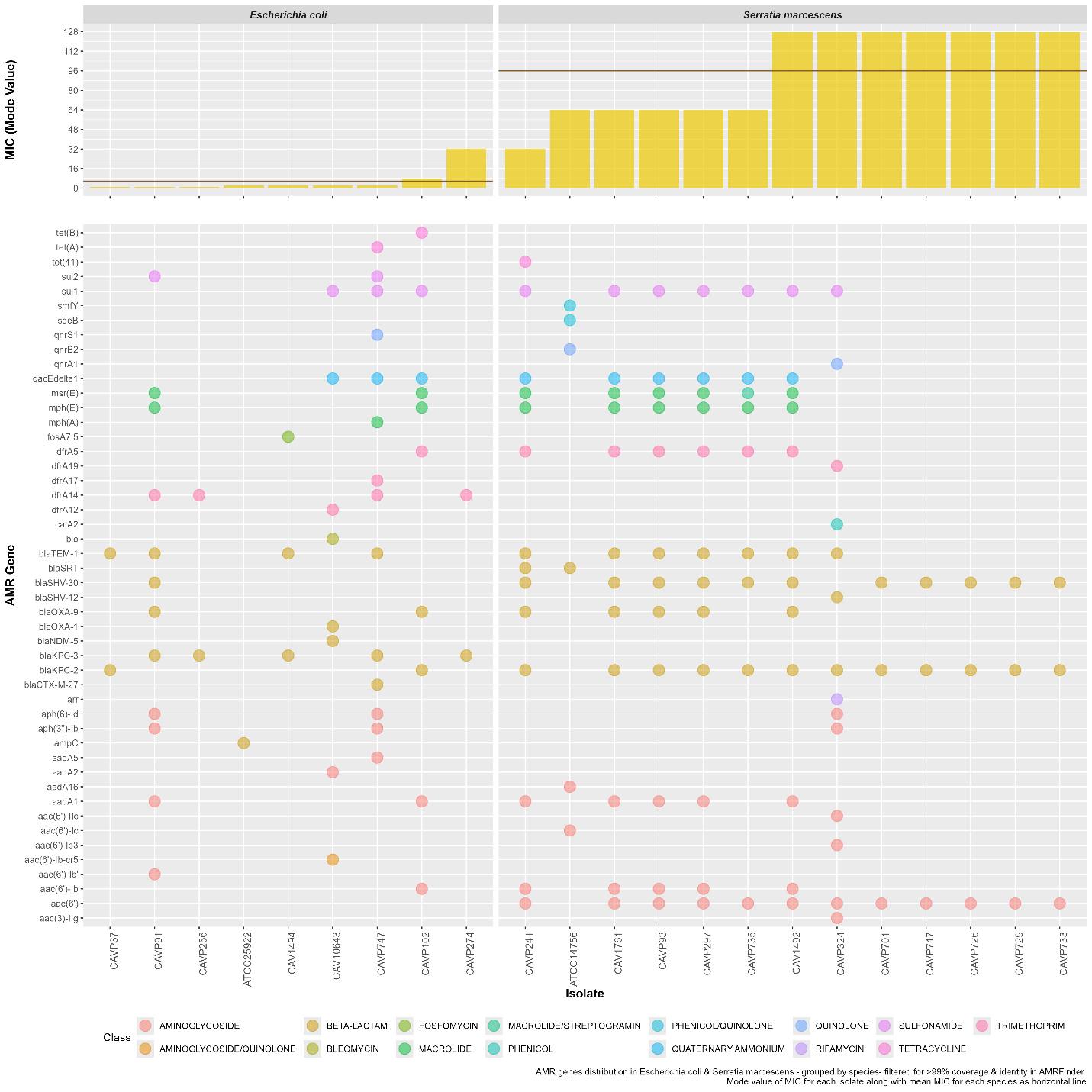


Supplemental Table S1. Point-biserial correlation analysis between the presence/absence of the qacEΔ1 gene and chlorhexidine MIC values across all species examined. No significant association observed.

| Species | estimate | statistic | p.value | parameter | conf.low | conf.high |
| --- | --- | --- | --- | --- | --- | --- |
| *Escherichia coli* | 0.00297 | 0.0214 | 0.983 | 52 | -0.265 | 0.271 |
| *Klebsiella aerogenes* | -0.103 | -0.462 | 0.649 | 20 | -0.503 | 0.333 |
| *Klebsiella grimontii* | NA | NA | NA | 11 | NA | NA |
| *Klebsiella oxytoca* | 0.0144 | 0.0945 | 0.925 | 43 | -0.28 | 0.307 |
| *Klebsiella pneumoniae* | 0.0371 | 0.52 | 0.604 | 196 | -0.103 | 0.176 |
| *Klebsiella quasipneumoniae* | 0.0527 | 0.796 | 0.427 | 227 | -0.0774 | 0.181 |
| *Klebsiella varicola* | NA | NA | NA | 47 | NA | NA |
| *Serratia marcescens* | -0.103 | -1.06 | 0.292 | 104 | -0.288 | 0.0892 |

Supplemental Table S2. Log2 MIC Results comparison using Dunn Statistical Test

| group1 | group2 | n1 | n2 | EstΔ | Est1 | Est 2 | statistic | p | p.adj | p.adj.signif |
| --- | --- | --- | --- | --- | --- | --- | --- | --- | --- | --- |
| *Escherichia coli* | *Klebsiella aerogenes* | 27 | 18 | 81.5 | 27 | 108.5 | 4.109174 | 3.97E-05 | 0.001429 | ** |
| *Escherichia coli* | *Klebsiella grimondii* | 27 | 3 | 104 | 27 | 131 | 2.621804 | 0.008747 | 0.314876 | ns |
| *Escherichia coli* | *Klebsiella michiganensis* | 27 | 12 | 49.25 | 27 | 76.25 | 2.177868 | 0.029416 | 1 | ns |
| *Escherichia coli* | *Klebsiella oxytoca* | 27 | 9 | 14 | 27 | 41 | 0.55804 | 0.576817 | 1 | ns |
| *Escherichia coli* | *Klebsiella pneumoniae* | 27 | 39 | 55.88 | 27 | 82.88 | 3.424676 | 0.000616 | 0.022159 | * |
| *Escherichia coli* | *Klebsiella quasipneumoniae* | 27 | 66 | 106.04 | 27 | 133.04 | 7.121795 | 1.07E-12 | 3.84E-11 | **** |
| *Escherichia coli* | *Klebsiella variicola* | 27 | 15 | 99.5 | 27 | 126.5 | 4.740356 | 2.13E-06 | 7.68E-05 | **** |
| *Escherichia coli* | *Serratia marcescens* | 27 | 45 | 178 | 27 | 205 | 11.2183 | 3.32E-29 | 1.19E-27 | **** |
| *Klebsiella aerogenes* | *Klebsiella grimondii* | 18 | 3 | 22.5 | 108.5 | 131 | 0.553547 | 0.579889 | 1 | ns |
| *Klebsiella aerogenes* | *Klebsiella michiganensis* | 18 | 12 | -32.25 | 108.5 | 76.25 | -1.32764 | 0.184296 | 1 | ns |
| *Klebsiella aerogenes* | *Klebsiella oxytoca* | 18 | 9 | -67.5 | 108.5 | 41 | -2.53667 | 0.011191 | 0.402881 | ns |
| *Klebsiella aerogenes* | *Klebsiella pneumoniae* | 18 | 39 | -25.61 | 108.5 | 82.88 | -1.37917 | 0.167843 | 1 | ns |
| *Klebsiella aerogenes* | *Klebsiella quasipneumoniae* | 18 | 66 | 24.54 | 108.5 | 133.04 | 1.4162 | 0.156717 | 1 | ns |
| *Klebsiella aerogenes* | *Klebsiella variicola* | 18 | 15 | 18 | 108.5 | 126.5 | 0.789919 | 0.429575 | 1 | ns |
| *Klebsiella aerogenes* | *Serratia marcescens* | 18 | 45 | 96.5 | 108.5 | 205 | 5.308656 | 1.10E-07 | 3.98E-06 | **** |
| *Klebsiella grimondii* | *Klebsiella michiganensis* | 3 | 12 | -54.75 | 131 | 76.25 | -1.30129 | 0.193158 | 1 | ns |
| *Klebsiella grimondii* | *Klebsiella oxytoca* | 3 | 9 | -90 | 131 | 41 | -2.07118 | 0.038342 | 1 | ns |
| *Klebsiella grimondii* | *Klebsiella pneumoniae* | 3 | 39 | -48.11 | 131 | 82.88 | -1.23208 | 0.217921 | 1 | ns |
| *Klebsiella grimondii* | *Klebsiella quasipneumoniae* | 3 | 66 | 2.045 | 131 | 133.04 | 0.05316 | 0.957605 | 1 | ns |
| *Klebsiella grimondii* | *Klebsiella variicola* | 3 | 15 | -4.5 | 131 | 126.5 | -0.10916 | 0.913075 | 1 | ns |
| *Klebsiella grimondii* | *Serratia marcescens* | 3 | 45 | 74 | 131 | 205 | 1.903983 | 0.056912 | 1 | ns |
| *Klebsiella michiganensis* | *Klebsiella oxytoca* | 12 | 9 | -35.25 | 76.25 | 41 | -1.22644 | 0.220033 | 1 | ns |
| *Klebsiella michiganensis* | *Klebsiella pneumoniae* | 12 | 39 | 6.63 | 76.25 | 82.88 | 0.308346 | 0.757819 | 1 | ns |
| *Klebsiella michiganensis* | *Klebsiella quasipneumoniae* | 12 | 66 | 56.79 | 76.25 | 133.04 | 2.776604 | 0.005493 | 0.197748 | ns |
| *Klebsiella michiganensis* | *Klebsiella variicola* | 12 | 15 | 50.25 | 76.25 | 126.5 | 1.990561 | 0.046529 | 1 | ns |
| *Klebsiella michiganensis* | *Serratia marcescens* | 12 | 45 | 128.75 | 76.25 | 205 | 6.079834 | 1.20E-09 | 4.33E-08 | **** |
| *Klebsiella oxytoca* | *Klebsiella pneumoniae* | 9 | 39 | 41.88 | 41 | 82.88 | 1.737691 | 0.082265 | 1 | ns |
| *Klebsiella oxytoca* | *Klebsiella quasipneumoniae* | 9 | 66 | 92.04 | 41 | 133.04 | 3.974202 | 7.06E-05 | 0.002542 | ** |
| *Klebsiella oxytoca* | *Klebsiella variicola* | 9 | 15 | 85.5 | 41 | 126.5 | 3.111089 | 0.001864 | 0.067104 | ns |
| *Klebsiella oxytoca* | *Serratia marcescens* | 9 | 45 | 164 | 41 | 205 | 6.890639 | 5.55E-12 | 2.00E-10 | **** |
| *Klebsiella pneumoniae* | *Klebsiella quasipneumoniae* | 39 | 66 | 50.16 | 82.88 | 133.04 | 3.810302 | 0.000139 | 0.004997 | ** |
| *Klebsiella pneumoniae* | *Klebsiella variicola* | 39 | 15 | 43.61 | 82.88 | 126.5 | 2.202449 | 0.027634 | 0.994811 | ns |
| *Klebsiella pneumoniae* | *Serratia marcescens* | 39 | 45 | 122.11 | 82.88 | 205 | 8.56356 | 1.09E-17 | 3.94E-16 | **** |
| *Klebsiella quasipneumoniae* | *Klebsiella variicola* | 66 | 15 | -6.54 | 133.04 | 126.5 | -0.35108 | 0.725532 | 1 | ns |
| *Klebsiella quasipneumoniae* | *Serratia marcescens* | 66 | 45 | 71.95 | 133.04 | 205 | 5.710314 | 1.13E-08 | 4.06E-07 | **** |
| *Klebsiella variicola* | *Serratia marcescens* | 15 | 45 | 78.5 | 126.5 | 205 | 4.039531 | 5.36E-05 | 0.001928 | ** |

Estimate (Est)

Supplemental Table S3. Long read sequenced isolates in study and corresponding genomic location of potential Efflux Pumps

| **x** | **isolate_id** | **isolate_species** | **Long Read Sequenced** | **Method of LRS** | **Chromosome** | **Plasmids** |
| --- | --- | --- | --- | --- | --- | --- |
| 26 | CAVp37 | Escherichia coli | Yes | ONT | -- | -- |
| 27 | CAVp102 | Escherichia coli | Yes | ONT |  | tet(B), qacEdelta1 |
| 28 | CAVp91 | Escherichia coli | Yes | ONT | -- | -- |
| 31 | CAVp274 | Escherichia coli | Yes | ONT | -- | -- |
| 44 | CAV10643 | Escherichia coli | Yes | ONT |  | qacEdelta1 |
| 11 | CAVP110 | Klebsiella aerogenes | Yes | ONT | oqxA, oqxB | qacE, qacEdelta1 |
| 16 | CAVP637 | Klebsiella aerogenes | Yes | ONT | oqxA, oqxB |  |
| 18 | CAVP795 | Klebsiella michiganensis | Yes | ONT | oqxB |  |
| 35 | CAV1015 | Klebsiella oxytoca | Yes | PacBio | oqxA, oqxB | qacE, qacEdelta1 |
| 37 | CAV1193 | Klebsiella pneumoniae | yes | PacBio | oqxA, oqxB | qacE, qacEdelta1 |
| 40 | CAVP45 | Klebsiella pneumoniae | Yes | ONT | oqxA, oqxB20 | tet(A), qacEdelta1 |
| 43 | CAV1016 | Klebsiella pneumoniae | Yes | PacBio | oqxA11, oqxB19 | qacEdelta1 |
| 46 | CAVp744 | Klebsiella pneumoniae | Yes | ONT | oqxA, oqxB32 |  |
| 57 | CAVp740 | Klebsiella pneumoniae | Yes | ONT | oqxA, oqxB14 |  |
| 6 | CAV2013 | Klebsiella quasipneumoniae | Yes | PacBio | oqxA, oqxB | qacE, qacEdelta1 |
| 7 | CAV2018 | Klebsiella quasipneumoniae | Yes | ONT, PacBio | oqxA, oqxB | qacEdelta1 |
| 39 | CAVP8 | Klebsiella quasipneumoniae | Yes | ONT | oqxA, oqxB | qacE, qacEdelta1 |
| 45 | CAV10628 | Klebsiella quasipneumoniae | Yes | ONT | oqxA, oqxB | tet(A) |
| 64 | CAVP360 | Klebsiella quasipneumoniae | Yes | ONT | oqxA, oqxB | qacEdelta1 |
| 66 | CAVP186 | Klebsiella quasipneumoniae | Yes | ONT | oqxA, oqxB | qacEdelta1 |
| 69 | CAVP296 | Klebsiella quasipneumoniae | Yes | ONT | oqxA, oqxB | qacEdelta1 |
| 17 | CAVP777 | Klebsiella variicola | Yes | ONT | oqxA, oqxB9 |  |
| 19 | CAVP796 | Klebsiella variicola | Yes | ONT | oqxA, oqxB15 |  |
| 20 | CAVP797 | Klebsiella variicola | Yes | ONT | oqxA, oqxB15 |  |
| 2 | CAV1492 | Serratia marcescens | Yes | PacBiob | tet(41) | qacEdelta1 |
| 3 | CAV1761 | Serratia marcescens | Yes | PacBio | tet(41) | qacEdelta1 |
| 14 | CAVP297 | Serratia marcescens | Yes | ONT | tet(41) | qacEdelta1 |
| 48 | CAVp717 | Serratia marcescens | Yes | ONT | tet(41) |  |
| 49 | CAVp726 | Serratia marcescens | Yes | ONT | tet(41) |  |
| 51 | CAVp729 | Serratia marcescens | Yes | ONT | tet(41) |  |
| 54 | CAVp735 | Serratia marcescens | Yes | ONT | tet(41) | qacEdelta1 |
| 71 | CAVp722 | Serratia marcescens | Yes | ONT | tet(41) |  |
